# Unconventionally Designed Tracking Loop Adaptable to Plasma Sheath Channel for Hypersonic Vehicles

**DOI:** 10.3390/s21010021

**Published:** 2020-12-22

**Authors:** Lei Shi, Shurong Yuan, Bo Yao

**Affiliations:** 1 Key Laboratory of Equipment Efficiency in Extreme Environment, Ministry of Education, XiDian University, Xi’an 710071, China; sryuan@stu.xidian.edu.cn (S.Y.); yaob@xidian.edu.cn (B.Y.); 2 School of Aerospace Science and Technology, XiDian University, Xi’an 710071, China

**Keywords:** phase tracking loop, plasma sheath, amplitude attenuation, Kalman filter

## Abstract

An aircraft that moves through the atmosphere at hypersonic speed is covered by plasma sheath, which causes random and fast time-varying amplitude attenuation and phase fluctuation in received signals. This paper comprehensively analyzes the mechanism of the amplitude attenuation effects on a traditional phase-locked loop (PLL), which is always ignored in traditional scenarios (such as satellite telemetry and vehicle communication). Simulation results and theoretical analysis showed that traditional PLL does not work reliably for signal carrier tracking with the severe time-varying amplitude attenuation of the plasma sheath channel. In this paper, an unconventionally designed Kalman filter (KF) tracking loop that is aware of phase dynamics and amplitude attenuation fluctuation for hypersonic vehicles is proposed. To introduce time-varying amplitude attenuation into the proposed KF-based tracking loop, the amplitude attenuation is first modeled with an autoregressive model. The statistical characteristics of the amplitude and phase fluctuation are then incorporated into the state equation and observation equation. Simulation results indicate that the proposed tracking loop is stable when the signal-to-noise ratio is −10 dB with the Ka band, even in the most severe flight environment for hypersonic vehicles.

## 1. Introduction

An aircraft that moves through the atmosphere at hypersonic speed is enveloped by plasma sheath because of the shock wave that heats the surrounding air and causes ablation of the heat shield, thereby causing air molecules and heat shield material to be dissociated and ionized. The plasma sheath can strongly attenuate electromagnetic waves, which leads to severe amplitude attenuation and phase fluctuation in received signals, where amplitude attenuation has fast time-varying fluctuation and deep-fading characteristics [1,2,3,4,5,6]. In addition, the received signals are affected by large phase dynamics caused by the relatively hypersonic movement between the signal transmitter and receiver including the Doppler shift and Doppler frequency rate [7]. In the design of carrier tracking loops for hypersonic vehicles, in addition to considering the large Doppler dynamic, as in conventional tracking loop design, the time-varying amplitude attenuation and phase fluctuation of the received signal must also be considered. Thus, the implementation of a robust and adaptive carrier-tracking algorithm is crucial to guarantee reliable synchronicity in such severe propagation conditions.

The phase-locked loop (PLL) architecture designed for mass-market signal receivers consists of a PLL discriminator, a loop filter, a carrier generator, and a numerically controlled oscillator [8,9]. The loop filter always has a constant bandwidth with a fixed order. Owing to the fixed bandwidth, the receiver cannot mark a difference between noise suppression and phase dynamics. A tradeoff is always needed in bandwidth because noise reduction requires a low bandwidth whereas phase dynamics requires a large bandwidth. Thus, frequency lock loop (FLL)-assisted PLL is used to solve the bandwidth conflict between the high dynamics and the low signal-to-noise ratio (SNR) of received signals [10,11]. However, poor stability caused by frequent switching between FLL and PLL makes this method unsuitable for hypersonic vehicles. Generally, conventional PLLs work in a mild environment (such as GPS and GNSS receivers), where the deep fading and fast time-varying characteristics of the amplitude attenuation of received signals are not obvious. In such conditions, the amplitude attenuation of the received signal has a negligible effect on the performance of the PLL in traditional scenarios. However, for hypersonic vehicles, the fast fluctuation in the amplitude of the received signal is extremely severe under the dynamic plasma sheath channel (PSC). As a result, the output of the PLL discriminator does not represent the true phase difference change between the received signal and the local carrier. Moreover, the traditional PLL will frequently be out of lock, and cycle slips will occur because of deep fading and the high-dynamic of the PSC.

To address these problems and improve the performance of carrier phase tracking, in 2017, Zhu began to study the influence of phase noise on carrier synchronization under the plasma sheath channel [12]. This revealed the impact of the phase noise on carrier tracking for three typical TT&C signals at the Ka band, and a tracking loop based on traditional PLL was proposed [13]. However, the tracking loop in [13] is based on type3-PLL with fixed bandwidth of loop filter and although it can alleviate the degradation in the performance of the PLL caused by amplitude attenuation under the plasma sheath, it still cannot take into account the conflict between the large dynamic Doppler frequency and low SNR at the same time. Recently, high-dynamic carrier tracking algorithms based on digital processing have been widely explored. The most popular one involves the introduction of the Kalman filter into the PLL instead of the loop filter [14,15]. This approach can adaptively adjust the loop bandwidth and effectively balance the conflict between low SNR and the large dynamic of the received signal on loop filter bandwidth requirements. In addition, Kalman-filter-based PLL can track multiple variables, which allows it to consider the effect of the deep amplitude fluctuation of the received signal during the carrier-tracking process. Therefore, this paper designs an unconventional tracking loop based on the Kalman filter structure in a hypersonic harsh channel environment. The designed loop is unlike the traditional PLL tracking loop, which approximates the real phase difference according to the output of the phase detector. Instead, tracing the time-varying amplitude attenuation together with the phase fluctuation is introduced in the Kalman filter structure. Thus, the phase detector output is associated with the time-varying amplitude attenuation and the real phase difference of the received signal, which enables it to eliminate the effect of deep amplitude fluctuation on carrier tracking.

In this paper, a novel Kalman-filter-based tracking loop for hypersonic vehicles under time-varying deep-fading PSC is put forward to simultaneously track the carrier phase and fast time-varying deep-fading amplitude attenuation. The performance of the algorithm was analyzed when the carrier frequency was at the Ka band, C band, and S band. The main contributions of this paper are as follows.

1. The influence mechanism of the amplitude attenuation effects on the traditional PLL is considered, which is always ignored in traditional scenarios. The amplitude attenuation and phase fluctuation of received signals were analyzed at different typical flight altitudes. Simulation results and theoretical analysis showed that traditional PLL does not work reliably for signal carrier tracking with severe time-varying amplitude attenuation under PSC.
2. An unconventionally designed Kalman filter tracking loop for hypersonic vehicles under time-varying deep-fading PSC is proposed to simultaneously track the carrier phase and fast time-varying deep-fading amplitude attenuation. The estimated tracking of the true phase difference under the PSC does not depend on the observed value of the phase detector. The tracking can be based on the mathematical relationship between the real phase error of the output and the phase detector output, which can be obtained from the established Kalman filter. Hence, the problem of lock-out caused by the mathematical model in the traditional PLL tracking loop that does not match the actual tracking situation can be prevented.

The rest of this paper is organized as follows. Section 2 illustrates the fast time-varying amplitude attenuation and phase fluctuation characteristics of the received signal that are introduced by the plasma sheath under typical re-entry conditions. Section 2 also demonstrates that the traditional PLL tracking loop is likely to lose its lock under deep-fading PSC. Section 3 presents the designs of the new Kalman tracking loop, which is adaptable to the PSC, and the state equations and observation equations of the new Kalman filter are constructed. In Section 4, the performance of the proposed Kalman tracking loop is evaluated under typical frequency points that are commonly used in telemetry scenarios, that is, the carrier frequency is in the S band (2.3 GHz), C band (5.8 GHz), and Ka band (30 GHz). Section 5 provides the conclusion and discussion.

## 2. The Performance of Traditional PLL under PSC

### 2.1. Amplitude Attenuation and Phase Fluctuation Characteristics

Based on electromagnetic (EM) signal propagation, the plasma sheath is a complex random medium with a spatial-temporal disturbance [16]. However, when an electromagnetic wave that is much shorter than the variation time of its electron density passes through plasma, the electron density does not change from the time that the electromagnetic wave enters plasma until it passes through the plasma. Therefore, when the distribution of the plasma sheath electron density is known, a uniform stratified analytical solution can be used to calculate the amplitude attenuation and phase noise of the signal passing through the plasma [12].

The typical plasma sheath electron density *N_e_(z)* with spatial turbulence at different flight altitudes can be obtained from the RAM-C projects [17]. The general function of *N_e_(z)* can be expressed as [18]:

(1) $$N_{e}(z)=\left\{\begin{array}{l} n_{e,\max}\exp[-\sigma_{1}{\left(z-z_{B}\right)}^{2}](0<z<z_{B})\\ n_{e,\max}\exp[-\sigma_{2}{\left(z-z_{B}\right)}^{2}](z_{B}<z<z_{T}) \end{array}\right.,$$

where *σ*_1_ and *σ*_2_ are shape parameters, *n_e, max_* is the electron density peak value at a considerable distance from the vehicle, *z_T_* is the thickness of plasma sheath, and *z_B_* is the position of *n_e,max_*. The fluctuant electron density *N_e_(z,t)* of plasma sheath in the spatial-temporal domain can be established by adding the time term, which can be written as follows [13]:

(2) $$N_{e}(z,t)=N_{e}(z)*\left[1+\Delta *n(t)\right],$$

where *N_e_(z)* is the plasma sheath electron density with spatial turbulence, *z* is the axis normal to the vehicle surface, ∆ is the relative fluctuant intensity, and *n(t)* is a non-stationary colored Gaussian random process with unit standard deviation that presents the dynamic characteristics caused by fluid turbulence [13].

**Figure 1:**
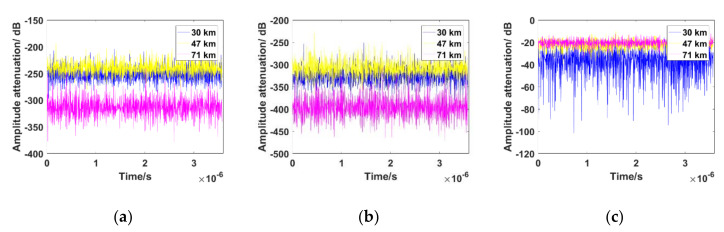
Amplitude attenuation of the received signal at different altitudes of RAM-C. (**a**) 2.3 GHz; (**b**) 5.8 GHz; (**c**) 30 GHz.

**Figure 2:**
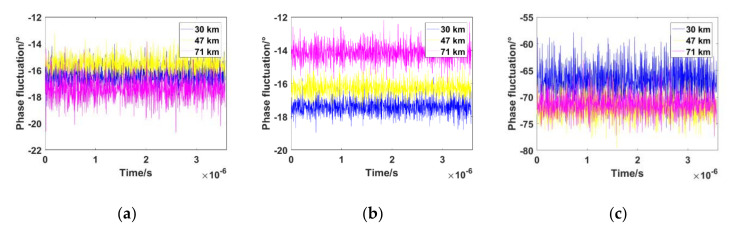
Phase fluctuation of the received signal at different altitudes of RAM-C. (**a**) 2.3 GHz; (**b**) 5.8 GHz; (**c**) 30 GHz.

To analyze the influence of plasma sheath on traditional PLL, the plasma sheath electron density at typical flight altitudes (30 km, 47 km, 71 km) was selected in the RAM-C program to calculate the amplitude attenuation and the phase fluctuation of received signals. The program contains real measurement data with regard to the combined effect of the trajectory, angle of attack, and velocity of the hypersonic vehicle. The parameters of the steady plasma sheath (*n_e, max_*, *z_T_*, *z_B_*, *σ_1_*, *σ_2_*) were set according to RAM-C measured data, with a relative fluctuant intensity ∆ = 10%. Figure 1 and Figure 2 show the simulation results of the amplitude attenuation and phase fluctuation of the received signal according to the algorithm mentioned in [18]. The carrier frequency was set to the S band (2.3 GHz), C band (5.8 GHz), and Ka band (30 GHz).

As shown in Figure 1a,b, when the carrier frequency was set to the S band and C band, which are commonly used for Unified S Band-Tracking Telemetry & Command (USB-TTC) telemetry, the amplitude attenuation of the received signals is almost as low as −400 dB under PSC. In contrast, the amplitude attenuation in the Ka band does not appear to be influenced by plasma sheath, which is approximately −20 dB, most of the time. However, in the worst circumstance under 30 km altitude [19], the calculated results show that deep fading may be −80 dB, which is caused by random time-varying turbulence even in the Ka band.

Figure 2 shows the phase fluctuation of the received signal caused by plasma sheath. The phase fluctuation in the S band and C band is much smaller than that in the Ka band. The statistical characteristics of the phase fluctuation subject to Gaussian distribution have been analyzed in [20].

### 2.2. The Impact of Time-Varying Amplitude Attenuation on Traditional PLL

With regard to the receiver of a telemetry system and considering signal distortion (amplitude attenuation and phase shift), the received signal is expressed as [12]:

(3) $$y(t_{n})=r(t_{n})\cdot A\cdot e^{j\theta_{t_{n}}}+n_{t_{n}},$$

where *r(t_n_)* is the amplitude attenuation caused by plasma sheath, *A* is the signal amplitude at transmitter and its value is 1 for simplification, and the carrier phase includes the phase variations due to the receiver’s dynamics and the phase fluctuation caused by plasma sheath, *n_tn_* is considered additive Gaussian white noise with a mean of 0 and a variance *σ_tn_*^2^, that is, *n_tn_* ~*N*(0, *σ_tn_*^2^).

The PLL architecture consists of a PLL discriminator, a loop filter, a carrier generator, and a numerically controlled oscillator. In the process of carrier tracking, the output of the PLL discriminator approximately represents the phase difference between the received signal and the local oscillator, and the oscillation frequency of the local oscillator can be adjusted in real time. When the carrier frequency of the input signal is larger than the local oscillator frequency, the output of PLL discriminator increases. As a result, the corresponding carrier frequency generated by the local oscillator increases, and the phase difference between the local carrier and the received signal decreases. Considering the time response and algorithmic complexity, the PLL discriminator generally consists of a multiplier and a low-pass filter. The specific output of the multiplier can be approximated by the signal shown in Equation (4).

(4) $$\theta_{e}(t_{n})=y(t_{n})\times U_{VCO},$$

where *y(t_n_)* is the received signal, *U_VCO_* is the local signal generated by the oscillator, *θ_e_(t_n_)* is the product of the local carrier and the received signal consisting of the high-frequency component and the low-frequency component. Equation (5) can be obtained by applying the low-pass filter to *θ_e_(t_n_)*. As shown in Equation (5), where the two outputs indicate that the in-phase and quadrature components of the received signal pass through the phase detector. When the amplitude attenuation *r_pll_(t_n_)* is equal to a constant value, the output phase detector *U_0_(t_n_)_pll_* approximately reflects the change of the real phase error ∆*θ_n_*.

(5) $$\Delta \theta_{n}\approx U_{0}{\left(t_{n}\right)}_{pll}=\left\{\begin{array}{l} r_{pll}(t_{n})\sin(\Delta \theta_{n})\\ r_{pll}(t_{n})\cos(\Delta \theta_{n}) \end{array}\right.,$$

Similarly, the output of the PLL discriminator under the PSC of a hypersonic vehicle *U_o_(t_n_)_plasma_* is shown in Equation (6).

(6) $$U_{0}{\left(t_{n}\right)}_{plasma}=\left\{\begin{array}{l} r_{pla}(t_{n})\sin(\Delta \theta_{n})\\ r_{pla}(t_{n})\cos(\Delta \theta_{n}) \end{array}\right.,$$

where ∆*θ_n_* is the real phase error, *r_pla_(t_n_)* is the amplitude attenuation of the received signal under the plasma sheath, which is shown in Figure 1. The deep fading of the amplitude attenuation of the received signal is serious and cannot be ignored whether it is in the S band, C band, or Ka band. The deep fading and fast time-varying characteristics of the PSC cause the value of *r_pla_(t_n_)* to fluctuate quickly and thus the output of the phase detector represents the overall variation caused by amplitude attenuation and the carrier phase, not just the amount of carrier phase variation as in traditional PLL.

**Figure 3:**
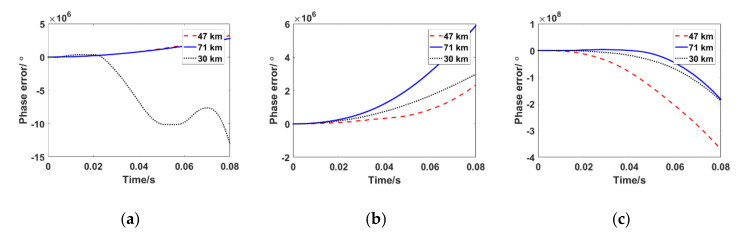
Phase error of the traditional phase-locked loop (PLL) under typical conditions of a time-varying plasma sheath channel (PSC). (**a**) 2.3 GHz; (**b**) 5.8 GHz; (**c**) 30 GHz.

To demonstrate the performance of traditional PLL under a typical PSC, illustrative simulation conditions were given as follows: the carrier frequency was set to the Ka band, C band, and S band. The Doppler shift of the received signal was 3 kHz, the Doppler frequency rate was 800 kHz/s, and the sampling period of the digitized signal Ts was 8 × 10^−6^ s. The values of the amplitude attenuation of the received signal under typical conditions (71 km, 47 km, 30 km) were used as shown in Figure 1. The tracking results of the traditional PLL under time-varying PSC are shown in Figure 3. The simulation results showed that when an aircraft is flying at 47 km, 71 km, and 30 km, traditional PLL cannot achieve stable carrier phase tracking. It is completely out of lock, whether the carrier frequency is at the Ka band, S band, or C band.

The simulation results are consistent with theoretical analysis. Conventional PLLs usually work in a mild environment, in which the time-varying characteristics of amplitude attenuation of the received signals are not obvious. In this case, the amplitude attenuation is regarded as an approximate fixed value; hence, the output of the phase detector only represents the amount of change in the true phase error. However, the phase of the received signal changes drastically because of the large dynamic Doppler in the hypersonic environment, which results in a large difference between the received signal and the local carrier. Simultaneously, the amplitude attenuation of the received signal has a fast time-varying characteristic due to the existence of the plasma sheath. Thus, the output of the phase discriminator reflects the overall change in amplitude attenuation and phase as shown in Equation (6). The output cannot be fed back to the real phase error in time, which causes the traditional PLL to lose its lock. A constant-bandwidth loop filter is essentially a smoothing and outlier rejection algorithm, which is only used to remove phase noise. Traditional PLL cannot solve the problem caused by deep amplitude attenuation with fast fluctuation.

## 3. Unconventionally Designed Kalman Filter Tracking Loop

**Figure 4:**
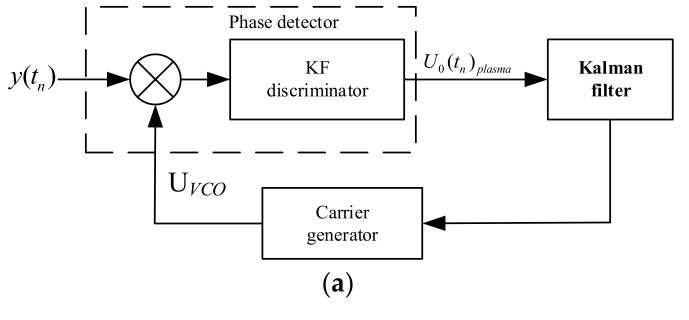

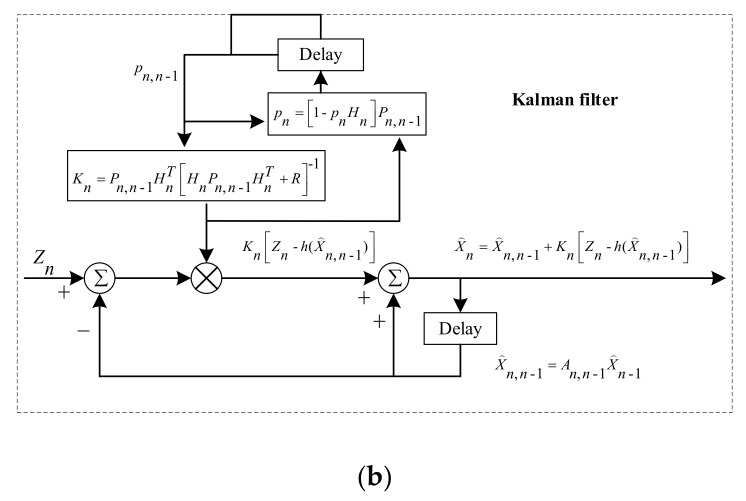
Unconventionally designed Kalman-filter-based carrier tracking loop. (**a**) Overall architecture and (**b**) Kalman filter specification.

As mentioned in the Section 2, the main problem of traditional PLL is the existing amplitude attenuation and noise reduction versus the dynamic range tradeoff, which may lose the lock. The PLL bandwidth is constant and a priori heuristically fixed by the user. However, the design and implementation simplicity of traditional PLL turns out to be its main drawback in deep fading and time-varying scenarios. In general, the gain of the Kalman filter is time-varying and optimally adjusted to the actual operation conditions at each time-step. It can track multiple variables, which makes it possible to simultaneously track the amplitude attenuation and phase of received signals. Thus, a Kalman filter is used to replace the loop filter of PLL in this paper. The architecture of the unconventionally designed Kalman filter tracking loop is shown in Figure 4a, where *y(t_n_)* is the received signal, *U_VCO_* is the local carrier generated by the oscillator, and *U_o_(t_n_)_plasma_* is the real-time output value of the phase detector. As shown in Figure 4a, the overall structure of the designed tracking loop is the same as that of traditional PLL except that the Kalman filter is designed to translocate the loop filter of traditional PLL. Section 3.1 describes the specific structure and related parameters of the Kalman filter illustrated in Figure 4b.

### 3.1. The Autoregressive (AR) Model of Amplitude Attenuation and the Statistical Characteristics of Phase Fluctuation in PSC

Under normal conditions, the Kalman filter is designed to establish the state-space formulation given in (7) and (8) for discrete-time systems [14].

(7) $$X_{n}=AX_{n-1}+BY_{n-1}+W_{n-1},$$

(8) $$Z_{n}=H_{n}X_{n}+V_{n},$$

where *A* is the transition matrix applied to pervious state *X_n−1_*, *B* is the control-input model applied to the control vector *Y_n−1_*, *Y_n−1_* can be described during the system state transition, *W_n−1_* is the process noise vector, *H_n_* is the measurement matrix, *V_n_* is the measurement noise, *Z_n_* is the measurement vector, and *X_n_* is the state vector.

The key step in designing a Kalman filter is to establish appropriate state-space equations. In general, traditional PLL based on a Kalman filter only considers the large dynamic Doppler of the signal phase; however, the state vector usually has three variables: the carrier phase, the Doppler shift, and the Doppler frequency rate [21,22]. As mentioned previously, the key problem for hypersonic vehicle carrier tracking is the amplitude attenuation and phase fluctuation of the received signals caused by the PSC. The system state-space equations considering the amplitude attenuation tracking to mitigate the effects of the PSC need to be re-established for the designed novel Kalman-filter-based tracking loop. The main function of the state equation is to use the value of the optimal state vector from the previous moment to estimate the state vector for the current moment, and the amplitude attenuation should be expressed in an iterative form. Therefore, estimation of the amplitude attenuation of received signals as a mathematical iterative formula is the basis for implementing a Kalman filter that considers amplitude attenuation and the carrier phase. Modeling signal amplitude attenuation with an AR model is a good way to meet the need for an iterative estimation in a state-space equation. However, the AR model is suitable for sequences with strong autocorrelation, and the error is extremely large when directly fitting the fast time-varying sequence to the AR model. Therefore, the mean value of the sequence is removed first as shown in Equation (9), and only the amount of fluctuation in the amplitude attenuation is autoregressively fit to reduce the error between the fitted value and the true amplitude attenuation.

(9) $$r^{\prime }(t_{n})=r(t_{n})-\mathrm{mean}\left[r(t_{n})\right],$$

The parameters of the AR model are given for the amplitude attenuation fluctuation after removing the mean value. To minimize model error, a second-order AR model is used as shown in Equation (10).

(10) $$r^{\prime }(t_{n})=\alpha_{2,1}r(t_{n-1})+\alpha_{2,2}r(t_{n-2})+v_{n},$$

where *v_n_* is a white Gaussian noise sequence with variance *σ_v_*. Coefficient *α*_2,1_, *α*_2,2_, and *σ_v_* can be obtained by using the Yule–Walker estimation method [23]. Table 1 shows the AR model parameters obtained by using the Levinson–Durbin algorithm at flight altitudes of 71 km, 30 km, and 41 km with a typical carrier frequency at the Ka band, C band, and S band, respectively.

Moreover, the statistical characteristics of the phase fluctuation caused by the PSC are analyzed in [20]. The statistical characteristics of the phase fluctuation demonstrate a Gaussian distribution. Table 2 shows the mean value, mean(*φ_n_*) and variance, *σ_φ,n_* of the phase fluctuation at flight altitudes of 71 km, 47 km, and 30 km.

### 3.2. The Design of Kalman-Filter-Based Tracking Loop

In the previous subsection, the AR model of amplitude attenuation fluctuation and the statistical characteristics of phase fluctuation in PSC were introduced. On this basis, the Kalman filter was designed to simultaneously track the amplitude attenuation and carrier phase error in the PSC. ∆*θ_n_* is the true phase error obtained by the value of the cross-correlation between the local oscillator carrier and received signal through a low-pass filter, which is established as Equation (11).

(11) $$\Delta \theta_{n}=\Delta \theta_{n-1}+T_{s}\times \omega d_{n-1}+\frac{1}{2}T_{s}^{2}\times \omega a_{n-1}+mean(\varphi_{n})+\upsilon_{\varphi ,n}+\upsilon_{1,n},$$

where ∆*θ_n−1_* is the phase error at the last moment, *Ts* is the time interval of tracking loop, *ωd_n-1_* is the Doppler shift, *ωa_n−1_* represents the drift rate of the Doppler shift, *φ_n_* = mean(*φ_n_*) + *υ_φ,n_* is the phase fluctuation of the received signal caused by the plasma sheath, and *υ_1,n_* is the noise generated in the circuits. According to Equation (11) and AR model Equation (10), the dynamic model of a discrete-time carrier phase processing under PSC can be developed by Equation (12).

(12) $$\left[\begin{array}{c} \Delta \theta_{n}\\ \omega d_{n}\\ \omega a_{n}\\ {r^{\prime }}_{n}\\ {r^{\prime }}_{n-1} \end{array}\right]=\left[\begin{array}{ccccc} 1 & T_{s} & \frac{1}{2}T_{s}^{2} & 0 & 0\\ 0 & 1 & T_{s} & 0 & 0\\ 0 & 0 & 1 & 0 & 0\\ 0 & 0 & 0 & \alpha_{2,1} & \alpha_{2,2}\\ 0 & 0 & 0 & 1 & 0 \end{array}\right]\left[\begin{array}{c} \Delta \theta_{n-1}\\ \omega d_{n-1}\\ \omega a_{n-1}\\ {r^{\prime }}_{n-1}\\ {r^{\prime }}_{n-2} \end{array}\right]+\left[\begin{array}{c} mean(\varphi_{n})\\ 0\\ 0\\ 0\\ 0 \end{array}\right]+\left[\begin{array}{c} \upsilon_{\varphi ,n}+\upsilon_{1,n}\\ \upsilon_{2,n}\\ \upsilon_{3,n}\\ \upsilon_{n}\\ 0 \end{array}\right],$$

Parameters *r′*, *α*_2,1_, *α*_2,2_, and *v_n_* have the same meaning as in Equation (10). *υ_2,n_* is the noise of Doppler shift and *υ_3,n_* is the noise of Doppler frequency rate. The Gaussian noise vector is defined as [*υ_φ,n_*+*υ_1,n_ υ_2,n_ υ_3,n_ υ_n 0_*]^T^, with diagonal covariance matrix Q = diag(*σ_φ,n_*+*σ_1,n_ σ_2,n_ σ_3,n_ σ_v_* 0).

*Z_n_* is the carrier phase error measurement, and it can be expressed as Equation (13), which is derived from the PLL’s in-phase and quadrature accumulations.

(13) $$Z_{n}=h(X_{n})+\left[\begin{array}{l} n_{i,n}\\ n_{q,n} \end{array}\right]=\left[\begin{array}{l} r_{n}\cos(\Delta \theta_{n})\\ r_{n}\sin(\Delta \theta_{n}) \end{array}\right]+\left[\begin{array}{l} n_{i,n}\\ n_{q,n} \end{array}\right],$$

where *n_i,n_* and *n_q,n_* are the noise at the in-phase and quadrature-phase output, respectively, of the discriminator, whose corresponding diagonal matrix is shown in Equation (14):

(14) $$R=E\left[V_{n}V_{n}^{T}\right]=\left[\begin{array}{cc} \frac{N_{0}}{2ST_{s}} & 0\\ 0 & \frac{N_{0}}{2ST_{s}} \end{array}\right],$$

where *V_n_* = [*n_i,n_ n_q,n_*]^T^ and *S/N_0_* is the carrier-to-noise density ratio [23]. In the Kalman filter formulation, instead of *h(X_n_)* as the measurement transition function, the linearized transition matrix (i.e., *H_n_* = ∇*h(X_n_)|*$X_{n}={\overset{⌢}{X}}_{n.n-1}$) in (15) is used.

(15) $$H_{n}=\left[\begin{array}{ccccc} \left({r^{\prime }}_{n}+mean(r(n)))\sin(\Delta \theta \right) & 0 & 0 & \cos(\Delta \theta ) & 0\\ -({r^{\prime }}_{n}+mean(r(n)))\cos(\Delta \theta ) & 0 & 0 & \sin(\Delta \theta ) & 0 \end{array}\right],$$

Then, the concrete form of the measurement equation can be written as Equation (16).

(16) $$Z_{n}=\left[\begin{array}{ccccc} \left({r^{\prime }}_{n}+mean(r(n)))\sin(\Delta \theta \right) & 0 & 0 & \cos(\Delta \theta ) & 0\\ -({r^{\prime }}_{n}+mean(r(n)))\cos(\Delta \theta ) & 0 & 0 & \sin(\Delta \theta ) & 0 \end{array}\right]\left[\begin{array}{c} \Delta \theta_{n}\\ \omega d_{n}\\ wa_{n}\\ r^{\prime }\\ {r^{\prime }}_{n-1} \end{array}\right]+\left[\begin{array}{l} n_{i,n}\\ n_{q,n} \end{array}\right],$$

As stated, the proposed state-space formulation allows the filter to be aware of amplitude attenuation variation and the carrier phase. According to the state-space formulation, the tracking algorithm (Algorithm 1) is given as follows:
**Algorithm 1. Tracking Algorithm**Require: $X_{0}$, $P_{0}=X_{0}X_{0}^{T}$, $Z_{n}$, Q, R(1) Set *n* = 1 (2) Estimate state: ${\overset{⌢}{X}}_{n,n-1}=A_{n,n-1}{\overset{⌢}{X}}_{n-1}$ (3) Estimate error covariance: $P_{n,n-1}=A_{n,n-1}P_{n-1}A_{n,n-1}^{T}+Q$ (4) Estimate the Kalman gain: $K_{n}=P_{n,n-1}H_{n}^{T}{\left[H_{n}P_{n,n-1}H_{n}^{T}+R\right]}^{-1}$ (5) Estimate the update state: ${\overset{⌢}{X}}_{n}={\overset{⌢}{X}}_{n,n-1}+K_{n}\left[Z_{n}-h({\overset{⌢}{X}}_{n,n-1})\right]$ (6) Estimate the corresponding error covariance: $P_{n}=\left[1-K_{n}H_{n}\right]P_{n,n-1}$ (7) Set *n = n+1* and repeat steps (2) to (7).

During the carrier tracking process, the frequency of the carrier generated by the local oscillator is updated as:

(17) $$w_{vco,n}=\omega d_{n-1},$$

where *ωd_n−1_* is the Doppler shift in the state vector estimated by the tracking algorithm. Similarly, the carrier phase is updated as:

(18) $$\theta_{vco,n}=\Delta \theta_{n-1}.$$

## 4. Results and Discussion

### 4.1. Performance Versus Carrier Frequency

**Figure 5:**
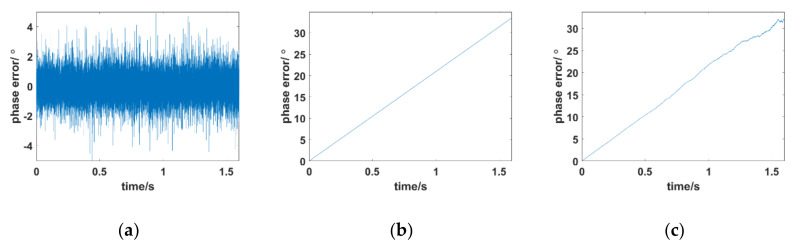
Phase error between the received signal and local carrier of the designed Kalman tracking loop at 30 km. (**a**) Ka band = 30 GHz; (**b**) C band = 5.8 GHz; (**c**) S band =2.3 GHz.

If the power of the telemetry communication link of an aircraft is sufficient, then the influence of the integrated link noise can be ignored, and only the influence of deep fading and fast time-varying attenuation on the carrier tracking algorithm of this paper should be of concern. We took 30 km, which is the worst condition for plasma, as an example to analyze the adaptability of the proposed method in S–Ka carrier tracking. Figure 5 provides the phase error between the received signal and local carrier of the designed Kalman tracking loop at the Ka band, C band, and S band. The Doppler shift in the received signal is 3 kHz, and the Doppler frequency rate is 800 kHz/s. The received signal only passes through the PSC without adding Gaussian white noise.

**Figure 6:**
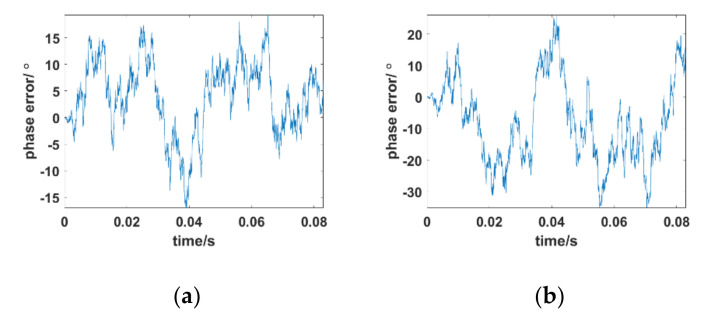
Phase error between the received signal and local carrier of designed Kalman tracking loop at 30 km with *λ = 10*^10^. (**a**) C band = 5.8 GHz; (**b**) S band = 2.3 GHz.

As illustrated in Figure 5a, when the carrier frequency is *f* = 30 GHz, the designed tracking loop can achieve stable tracking and the phase error of the novel tracking loop is between −3° and 3°. As the carrier frequency is reduced to the C band and S band, the phase error shows a linear growth trend, and the proposed tracking algorithm loses lock as shown in Figure 5b,c. This is because the amplitude attenuation of the received signals in the S band and C band is serious. The average value is as low as −300 dB and the fast time-varying characteristic is remarkable. The error between the AR model and the true amplitude attenuation is so large that the tracking loop cannot achieve stable tracking. To reduce the model error and increase the time variability of the AR model data fitting, a new correction factor *λ = 10*^10^ can be introduced to correct *σ_v_* to *σ_v_·λ* manually, where *λ* is an empirical parameter, which is found by the accumulation algorithm. The specific steps are described in Algorithm 2. Figure 6 shows the tracking results after substituting *new*(*σ_v_*) = *σ_v_*·*λ* in the AR model.

After adding correction factor *λ*, the iteration form of time-varying in the AR model is improved; hence, the proposed algorithm can achieve carrier tracking even in the S band. Generally, the smaller the error when converting the amplitude attenuation of the received signal to an iterative form, the better the algorithm performance. However, how to properly set the value of *λ* during the tracking process to improve algorithm performance in different tracking situations is a new challenge.
**Algorithm 2. The algorithm of setting *λ***1. Set the threshold of phase error of tracking loop, *k* = ±20°; Set initial value of λ, λ_0_ = 1; Set initial value of phase error *pe_0_* = 180°. The phase error of the tracking loop obtained in the i-th iteration is expressed as *pe_i_*2. While *pe_i_* > *k*
Increase λ; Get *pe_i_* by tracking loop; 3. Get λ

### 4.2. Performance Versus Flight Altitude (Average Attenuation) under the Ka band

**Figure 7:**
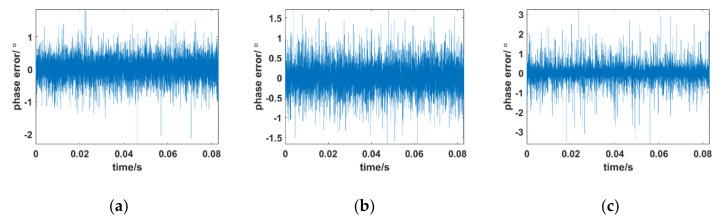
Phase error between the received signal and local carrier of the designed Kalman tracking loop at different flight altitudes. (**a**) 71 km (**b**) 47 km (**c**) 30 km.

Amplitude attenuation of aircraft flight at different altitudes corresponds to different characteristics. Figure 7 shows the phase error between the received signal and the local carrier of the designed Kalman tracking loop at different flight altitudes (71 km, 47 km, and 30 km) under the Ka band. Other simulation conditions are consistent with Section 4.1. In previous calculations, the average amplitudes of the received signal under plasma sheath of 71 km, 47 km, and 30 km are −17.4 dB, −18.4 dB, and −28.4 dB, respectively

Figure 7 shows the influence of amplitude attenuation of the received signal effects on the novel tracking loop. Figure 7a,b shows that when the aircraft is flying at 71 km and 47 km, the phase error remains at almost zero degrees with few fluctuations within 1.5 °C. As the average amplitude attenuation becomes increasingly serious under 30 km (Figure 7c), the phase error of the tracking loop gradually increases and the performance of the tracking loop slightly deteriorates. In typical data collected in the RAM-C Program, a 30 km flight altitude is almost the worst circumstance. The amplitude attenuation of the received signal is as low as −90 dB in the worst circumstance due to the existence of the plasma sheath. The phase error of the tracking loop is between −3° and 3°. Under this condition, the tracking loop can still achieve stable tracking with an acceptable degradation in performance.

### 4.3. Performance Versus Noise under the Ka band

**Figure 8:**
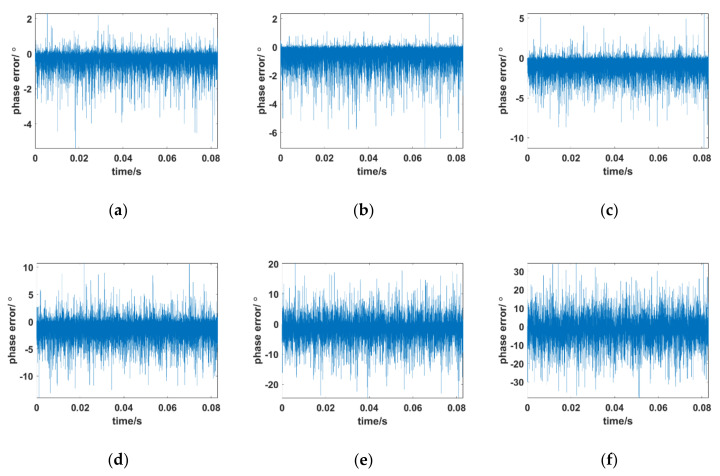
Phase error between the received signal and local carrier of the designed Kalman tracking loop at different signal-to-noise ratios (SNRs). (**a**) 10 dB; (**b**) 5 dB; (**c**) 0 dB; (**d**) −5 dB; (**e**) −10 dB; (**f**) −15 dB.

The previous simulations consider the signal passing through the PSC, and the results show that the proposed method can basically adapt to the influence of PSCs at different altitudes in the Ka band. However, when the integrated link margin is not sufficient, the influence of integrated link noise on the proposed algorithm must be considered. To emulate the practical scenario, Gaussian white noise is added to the received signal. Figure 8 shows the variation in the phase error of the designed tracking loop when the SNR changes from 10 dB to −15 dB at 30 km under the Ka band. Other simulation conditions are consistent with Section 4.1.

Figure 8 shows the phase error curves of the tracking loop versus the effect of the SNR. As SNR decreases, the tracking phase error increases gradually. Figure 8c shows that when the value of SNR drops to 0 dB, the phase error of the tracking loop can still work effectively. When the SNR continues to drop to −10 dB, the corresponding phase error is between 20° and −20°, which can still be tracked within the error range. A phase error close to ±30° and unreliable tracking accuracy may occur when the SNR drops to −15 dB. Compared with the carrier tracking loop described in [18], which only focuses on solving the problem of loss-of-lock caused by amplitude attenuation under the plasma sheath without considering large dynamic Doppler and Doppler acceleration, the tracking loop proposed in this paper not only works at low SNR but it also simultaneously takes the large dynamic Doppler into account.

Overall, the above analyses indicate the following implications. (1) Owing to the deep fading and fast time-varying effects of the plasma sheath, the traditional tracking loop always ignores the influence of amplitude attenuation and only tracks the phase characteristics, which makes it basically impossible to achieve effective carrier tracking in the S–Ka band. (2) When the SNR of the integrated link is sufficient, the designed Kalman filter tracking loop can track effectively under the plasma sheath of the Ka band carrier in different states. Although tracking performance decreases with the severity of the sheath, it is acceptable. (3) The noise impact of the integrated link needs to be considered when the SNR of the integrated link is limited. Fortunately, stable carrier tracking can be achieved when the SNR is higher than −10 dB at the Ka band with the proposed scheme. (4) Although carrier tracking of the S band and C band easily loses lock, the lockout threshold is potentially alleviated if correction factor λ can be reasonably adjusted in the AR model.

## 5. Conclusions

Hypersonic vehicles operate in a complicated environment, where received signals are affected by amplitude attenuation and phase fluctuation caused by plasma sheath and Doppler shift because of the hypersonic flight environment. A major problem is that traditional PLL tracking loop cannot remain locked in a hypersonic environment, therefore the influence mechanism of the amplitude attenuation effect on traditional PLL was considered. An unconventionally designed Kalman-filter-based tracking loop for hypersonic vehicles under time-varying deep-fading PSC was proposed to simultaneously track the carrier phase and fast time-varying deep fading amplitude attenuation. The performance of the algorithm was analyzed when the carrier frequency was in the Ka band, C band, and S band. Simulation results indicate that the designed tracking loop is stable when the SNR is −10 dB under the most severe flight environment for hypersonic vehicles with the Ka band. However, in view of the out-of-lock phenomenon of the designed tracking loop in the C band and S band, the correction factor *λ* needs to be automatically adjusted to achieve stable tracking, which is a new requirement that should be considered in the future.

## Figures and Tables

**Table 1:** Value of parameters in the autoregressive (AR) model.

Carrier Frequency/GHz	Flight Altitude/km	*α*_2,1_	*α*_2,2_	*σ_v/_*dB	Mean (r(tn))/dB
Ka band (30)	71	−0.3913	−0.4320	−65.7128	−17.4
	47	−0.3887	−0.4046	−63.1461	−18.4
	30	−0.3342	−0.3591	−69.0776	−28.4
C band (5.8)	71	−0.0116	−0.0117	−677.6254	−313.4
	47	−0.0072	−0.0075	−522.6127	−247.9
	30	−0.0115	−0.0116	−560.0640	−262.1
S band (2.3)	71	−0.0160	−0.0164	−545.6845	−254.4
	47	−0.1054	−0.1062	−446.4157	−200.3
	30	−0.0956	−0.0959	−462.1327	−208.8

**Table 2:** Mean value and variance of the phase fluctuation.

Carrier Frequency/GHz	Flight Altitude/km	*σ_φ,n_*	Mean (*φ_n_*)
Ka band (30)	71	4.4576	−71.3
	47	5.1877	−71.9
	30	10.2987	−66.9
C band (5.8)	71	0.3328	−14.1602
	47	0.2067	−16.3
	30	0.2221	−17.4
S band (2.3)	71	0.8437	−17.4
	47	0.4507	−15.5
	30	0.5305	−16.4302

## Data Availability

Data sharing not applicable. No new data were created or analyzed in this study. Data sharing is not applicable to this article.
